# What Is the Influence of Simulation-Based Training Courses, the Learning Curve, Supervision, and Surgeon Volume on the Outcome in Hernia Repair?—A Systematic Review

**DOI:** 10.3389/fsurg.2018.00057

**Published:** 2018-09-28

**Authors:** Ferdinand Köckerling

**Affiliations:** Department of Surgery and Center for Minimally Invasive Surgery, Academic Teaching Hospital of Charité Medical School, Vivantes Hospital, Berlin, Germany

**Keywords:** hernia, training course, learning curve, case load, supervision

## Abstract

**Introduction:** In hernia surgery, too, the influence of the surgeon on the outcome can be demonstrated. Therefore the role of the learning curve, supervised procedures by surgeons in training, simulation-based training courses and surgeon volume on patient outcome must be identified.

**Materials and Methods:** A systematic search of the available literature was carried out in June 2018 using Medline, PubMed, and the Cochrane Library. For the present analysis 81 publications were identified as relevant.

**Results:** Well-structured simulation-based training courses was found to be associated with a reduced perioperative complication rate for patients operated on by trainees. Open as well as, in particular, laparo-endoscopic hernia surgery procedures have a long learning curve. Its negative impact on the patient can be virtually eliminated through consistent supervision by experienced hernia surgeons. However, this presupposes availability of an adequate trainee caseload and of well-trained hernia surgeons and calls for a certain degree of centralization in hernia surgery.

**Conclusion:** Training courses, learning curve, supervision, and surgeon volume are important aspects in training and outcomes in hernia surgery.

## Introduction

Using multivariable analyses and propensity score-matched comparisons it is possible to identify the influence factors impacting the outcome in hernia surgery (1, 2). In hernia surgery, too, the influence of the individual surgeon on the outcome can be demonstrated (3). There is one prominent example of that in the literature. A Swedish surgeon not only impacted comparison of totally extraperitoneal patch plasty (TEP) vs. the Lichtenstein operation in primary inguinal hernia repair due to a high recurrence rate in a prospective randomized trial to the disadvantage of TEP but also impacted a meta-analysis, likewise to the disadvantage of TEP (4–6). A further aspect is that hernia surgery has become increasingly more complex due to the introduction of new techniques and technologies (7). That gives rise to a debate about appropriate training in hernia surgery (7). “Many studies have indicated that surgical trainees are not receiving sufficient experience, and are failing to reach nationally identified targets” (8). Therefore well-structured training opportunities and training concepts that take account of the learning curve, simulation-based training, supervision, surgeon, and hospital caseload are needed. The following analysis of the available literature investigates these aspects and their impact on outcomes in hernia surgery.

## Materials and methods

A systematic search of the available literature was performed in June 2018 using Medline, PubMed, and the Cochrane Library. Furthermore, surgical journals and the reference lists of published articles were searched for relevant studies.

The following search terms were used: “Hernia and learning curve,” “Hernia and training,” “Hernia and supervision,” “Hernia and training course,” “Hernia and caseload,” “Hernia and volume,” “Hernia and experience,” “Hernia and education,” “Hernia and simulation-based training courses.”

**Figure 1:**
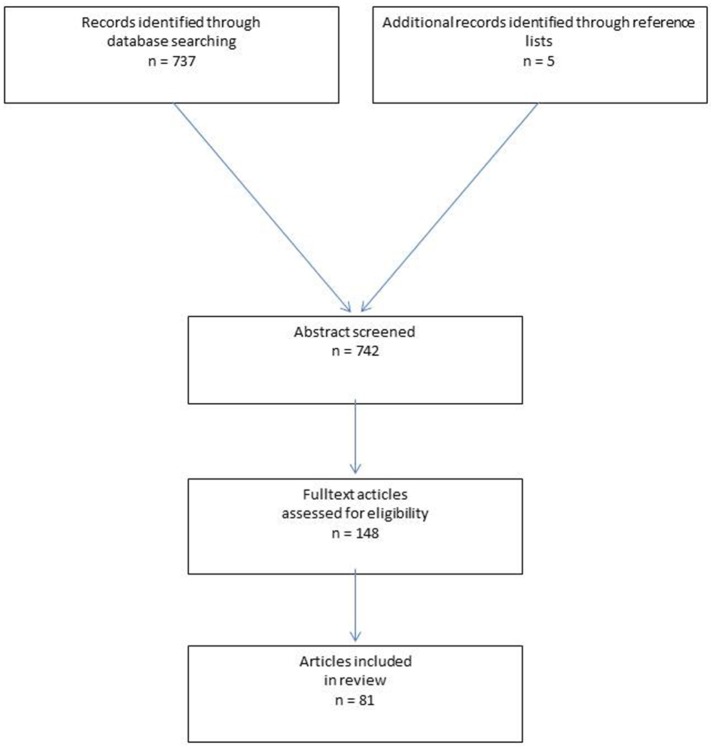
Flowchart of study inclusion.

The abstracts of 742 publications were consulted and a decision was taken on their inclusion in this literature review. For the present analysis 81 publications were identified as relevant (Figure 1). The quality of evidence according to Grade is moderate.

## Simulation-based training courses

“Surgical training has traditionally been an apprenticeship, where the surgical trainee learns to perform surgery under the supervision of a trained surgeon” (9). Based on the published guidelines, the techniques currently recommended for inguinal hernia repair are the laparo-endoscopic TEP (totally extraperitoneal patch plasty) and TAPP (transabdominal preperitoneal patch plasty) as well as the Lichtenstein technique, and for ventral and incisional hernia repair the laparoscopic IPOM (intraperitoneal only mesh), sublay and posterior component separation technique (6, 10–20). The learning curve in laparo-endoscopic techniques in hernia surgery is longer due to the greater complexity (11, 12). Hence, there is a growing call for the introduction of preclinical courses to help master the learning curve, especially in laparo-endoscopic hernia surgery (6, 11).

In the International guidelines for groin hernia management the recommendation for a goal-directed curriculum including anatomy, procedure steps, intraoperative decision-making and proficiency-based, simulation-enhanced technical skills training has been strongly upgraded (11).

In a systematic review about the state of evidence on simulation-based training for laparoscopic surgery significant effects were identified for simulation-based training courses for knowledge, skills time, skills process, skills product, behavior time, behavior process, and patient effects (21). And in an extended review of patient outcomes in simulation-based medical education an association was found between simulation-based education and small to moderate patient benefits (22). Here, too little attention has been paid to date to the cost aspect, whereby simulation-based medical education could have potential savings' effects (23). That applies to the costs incurred for training young surgeons as well as to the operative times and hospital stay (9).

In a prospective randomized trial (RCT) it was found that for inguinal hernia surgery in TEP technique a simulation-based mastery learning course led to a reduction in the operative time, improved trainee performance, a reduced intra- and postoperative complication rate and a lower hospital admission rate (24). Evaluation of simulation-based training courses for laparo-endoscopic hernia surgery showed that they received a very positive assessment from young surgeons in training (25).

Consensus recommendations from the Association of Surgeons in Training for improving the future of surgical training include a recommendation whereby trainees have an obligation to ensure they play a proactive role in utilizing all training opportunities available, including surgical simulation facilities (26).

These simulation-based training courses could also be part of a standardized curriculum concept for continuing training in hernia surgery along the lines of a “Hernia School” (7). In any case the goal should be, through the formulation of a definitive curriculum for acquisition of surgical skills outside the operating room, to prepare young surgeons for clinical surgical practice (27).

The corresponding concepts and models are available for hernia surgery (7, 24, 25, 28–31). Surgeons in training should be urgently required to intensively engage with these training concepts and training models before carrying out their initial procedures on hernia patients, in their own interest and that of the patient. The available state of evidence supports that demand but it should be further improved in future studies. However, that demand has already now been accordingly upgraded in the new International Guidelines for Groin Hernia Management, even in the absence of strong evidence so far, because of its implications for patient treatment (11).

## Learning curve

“Increasing performance through learning and repeating is well known” (32). “The repetition of a special task over a period of time leads to improvement of the results and shortens the time used to complete the task” (32). “In surgery the term ‘learning curve' is often used to describe the phenomenon of acquiring the surgical skills to perform a specific operation safely, sufficiently and effectively” (32). Since the learning curve in laparo-endoscopic hernia surgery is longer compared with in open procedures because of the greater complexity of the procedures (12), in the literature there are essentially more studies reporting on the learning curve in laparo-endoscopic compared with open hernia surgery. Likewise, since there seems to be also a difference between the learning curves in the endoscopic TEP and the laparoscopic TAPP in inguinal hernia repair, far more studies have been carried out on TEP (33–49) than on TAPP (50–52) (Tables 1, 2).

**Table 1:** Learning curve of inguinal hernia repair in totally extraperitoneal patch plasty (TEP) technique.

**References**	**Procedure time**	**Complication rate**	**Conversion rate**	**Recurrence rate**
Liem (33)	Mean procedure time: Cases 1–10 75 min Cases 11–20 68 min Cases 21–30 55 min *p* = 0.003	–	–	–
Wright (34)	Mean procedure time: Cases 1–10 75 min Cases 11–20 75 min Cases 21–30 60 min	–	Cases 1–10 20% Cases 11–20 20% Cases21–30 3%	–
Aeberhard (35)	Mean procedure time: Cases 1–15 105 min Cases 16–50 102 min Cases 51–100 84 min Cases 100 and more 53 min *p* = 0.001	–	–	–
Feliu-Pala (36)	–	Cases 1–100 33% Cases 101–500 5.25% Cases 501–1227 3.44% *p* < 0.01	Cases 1–100 17% Cases 101–200 12% Cases 201–500 5.3% Cases 501–750 2.4% Cases 751–1227 2.2% *p* < 0.01	Cases 1–100 14% Cases 101–500 1.5% Cases 501–1227 0.4% *p* < 0.01
Lau (37)	The mean procedure time reached a plateau value of < 1 h after performing 80 procedures	–	–	–
Neumayer (38)	–	–	–	Cases 1–250 10% Cases > 250 5% *p* < 0.01
Lal (39)	–	–	Cases 1–10 50% Cases 11–20 0% Cases 21–30 0% Cases 31–56 2%	–
Lamb (40)	–	–	–	Cases 1–20 10% Cases 21–80 4% Cases 81–200 2% Cases > 200 1%
Lim (41)	Mean procedure time: Cases 1–30 65 min Cases >30 52 min *p* = 0.015	Cases 1–30 20% Cases > 30 8.3%	–	–
Choi (42)	The mean duration of surgery significantly decreased (*p* < 0.001) in relation to experience; it reached a plateau of < 30 min (mean 28 min) after 60 Cases	–	–	–
Malik (43)	Mean procedure time: Cases 1–30 95 min Cases > 30 60 min	–	Cases 1–30 20% Cases > 30 0%	Cases 1–30 30% Cases > 30 0%
Shouten (44)	Mean procedure time: Cases 50–100 30 min Cases > 900–1,000 20 min	Cases 50–100 11.6% Cases > 900–1,000 4.2% *p* < 0.001	Cases 50–100 1.6% Cases > 900–1,000 0.2% *p* < 0.018	Cases 50–100 0.61% Cases > 900–1,000 0.11% *P* < 0.001
Park (45)	Mean procedure time: Cases 1–30 50 min Cases > 30 36 min	Cases 1–30 23.3% Cases > 30 15.4%	Cases 1–30 5% Cases > 30 1.9%	–
Hasbahceci (46)	Mean procedure time: Cases 1–21 58 min Cases 22–42 53 min	–	Cases 1–21 33.3% Cases 22–42 0% *p* = 0.009	–
Gupta (47)	Mean procedure time: Cases 1–25 116 min Cases 26–45 86 min	–	Cases 1–25 8% Cases 26–45 5%	Cases 1–25 8% Cases 26–45 5%
Mathur (48)	CUSUM analysis suggested an inflection point at 18 Cases after which operative time stabilized	–	–	–
Sugita (49)	After an initial reduction, the mean operating time stabilized after 65 Cases	–	–	–

**Table 2:** Learning curve of inguinal hernia repair in transabdominal preperitoneal patch plasty (TAPP) technique.

**References**	**Procedure time**	**Complication rate**	**Conversion rate**	**Recurrence rate**
Voitk (50)	–	Cases 1–50 16% Cases 51–100 8% *p* < 0.063	Cases 1–50 5% Cases 51–100 0% *p* < 0.05	Cases 1–50 5% Cases 51–100 0% ns
Edwards (51)	–	Cases 1–30 11.7% Cases > 30 0%	Cases 1–30 2.2% Cases > 30 1.2%	Cases 1–30 12.2% Cases > 30 0%
Bracale (52)	The procedure time became stabilized after 65 operations	–	–	–

The operative time for TEP is < 1 h once the surgeon has performed 30–100 operations (33, 37, 41–46). The postoperative complication rate can still be significantly reduced after more than 100 TEP operations compared with up to 100 operations (36, 44). That is also true for the conversion rate. Likewise, the recurrence rate can be significantly reduced after more than 250 TEP operations compared with up to 250 TEP operations (38).

Accordingly, the learning curve in the TEP technique for inguinal hernia repair, when taking into account all outcome criteria, spans a surgical volume of up to 250 operations. Hence, the TEP technique seems to be associated with a longer learning curve for inguinal hernia repair (Tables 1, 2), although some studies report about a much shorter learning curve (34, 39, 43, 45, 46).

For TAPP the learning curve is reported to be around 50–100 procedures (Table 2). As such, the learning curve in TAPP seems to be associated with a lower caseload compared with TEP. This might be explained by better comparability of TAPP with other laparoscopic operations and the much narrower spatial conditions in TEP. In a Consensus Development Conference of the European Association of Endoscopic Surgery a statement is given, that studies showed significant reduction of operating time, conversion rates, and complication rates after 30–100 TEP procedures and 50–75 TAPP procedures (12). In the new international guidelines for groin hernia management (11) no difference in the learning curve between TEP and TAPP was found.

Nevertheless, training in TEP and TAPP requires a corresponding caseload in the training hospital and longer supervision of trainees by an experienced laparo-endoscopic surgeon. This of courses means that higher costs are incurred for training in laparo-endoscopic surgery (23).

The longer learning curve in laparo-endoscopic surgery could possibly be reduced through more the provision of more intensive preclinical training courses with simulation models. Therefore further models must be developed for learning the laparo-endoscopic techniques and shortening the learning curve, and these should be made available in intensive preclinical training courses.

In open mesh repair of inguinal hernias unsupervised junior trainees had unacceptably high recurrence rates (53). On average, the trainees in a UK study achieved proficiency for independent inguinal hernia repair after they had performed 64 repairs (range 12–73) which usually was reached in their fourth year of training (11, 54).

Accordingly, open inguinal hernia repair with mesh also has a relevant learning curve. Therefore the benefits of structured, simulation-based training courses should also be exploited for open mesh repair of inguinal hernia.

The learning curve in laparoscopic ventral hernia repair is associated with a significantly higher intestinal injury rate on comparing the first 32 operations with the subsequent 32 operations (12 vs. 0%; *p* = 0.02) (55). Likewise, there were clear, but not significant, differences in the conversion rate (12 vs. 0%; *p* = 0.11) (55).

A further study of the learning curve in laparoscopic ventral hernia repair that identified a conversion rate of 13.8% and intestinal injury rate of 6.9% attests to a high complication rate during the learning curve (56).

An investigation of the learning curve in laparoscopic ventral hernia repair found that three experienced laparoscopic surgeons reached a plateau operative time after 12 operations each (57).

Here too, as pointed out above, the learning curve can be reduced though structured simulation-based training courses (55).

But mentoring and supervision by a surgeon experienced in this technique is crucial during the learning curve. Therefore the learning curve under the supervision of an experienced hernia surgeon is now analyzed in the following.

## Learning curve under supervision

Training in complex laparoscopic procedures under the supervision of an experienced surgeon can be performed safely without jeopardizing the patient's outcome (58). Only very rarely does a surgeon in training perform operations without the supervision of an experienced surgeon (59). The degree of supervision needed by a surgeon in training can apparently be well estimated by experienced surgeons (59). Appropriate supervision of surgeons in training does not lead to poorer patient outcomes (59).

In hernia surgery, too, there are a number of studies that investigated the role of supervision of surgeons in training or during the learning curve on these outcomes (32, 53, 60–66) (Table 3). Follow-up from 3.5 to 6.1 years of TEP operations carried out by trainees under the supervision of experienced consultants identified recurrence rates of 1% and 2.6%, respectively (60, 61). The chronic pain rate following TEP repair was 1.5% after 6.1 years (61).

**Table 3:** Results of supervised surgical training in hernia surgery.

**References**	**Hernia type**	**Procedure**	**Procedure time**	**Complication rate**	**Conversion rate**	**Recurrence rate**	**Chronic pain rate**
**A**							
Haidenberg (60)	Inguinal	TEP	–	–	–	1% with a mean follow-up of 3.5 years	–
Zendejas (61)	Inguinal	TEP	–	–	–	2.6% with a mean follow-up of 6.1 years	1.5% with a mean follow-up of 6.1 years
Bökeler (62)	Inguinal	TAPP	Mean: 59 min	3.2% with no significant difference to experienced surgeons	–	0.4% with no significant difference compared to experienced surgeons	–
Robson (53)	Inguinal	Open suture, open mesh, laparoscopic mesh	–	–	–	Supervised junior trainees had similar recurrence rates to consultants	–
Wiese (32)	Inguinal	Lichtenstein	Median 69 min	–	–	–	–
Koizumi (63)	Inguinal	Lichtenstein	Average operating time was 80.7 ± 24.9, 72.6 ± 20.8, 63.5 ± 20.0, and 54.7 ± 27.9 for junior residents, senior residents, junior faculty and senior faculty, respectively with a significant difference between junior residents and senior faculty (*p* < 0.001)	No significant difference	–	–	–
**B**							
El-Sharkawy (64)	Inguinal, umbilical, epigastric, incisional	Open and laparoscopic	No significant difference in procedure time between consultants and trainees	No difference in the death rate	–	–	–
Kahn (65)	Incisional	Open and laparoscopic	Operating time of supervised trainees compared to consultants similar (60 ± 4 min vs. 58 ± 4 min)	Trainees 20% vs. consultants 17% (*p* = 0.41)	–	Trainees 8% vs. consultants 16% (*p* = 0.22)	–
Brown (66)	Hiatal hernia with primary reflux disease	Laparoscopic	Trainees 70 (20–248) min vs. consultants 60 (20–270) min *p* < 0.0001	Trainees 11% vs. consultants 9% (*p* = 0.197)	–	Reasons for reoperations were dysphasia (2.6% for consultants vs. 4.5% for trainees; *p* = 0.045), paraesophageal hernia (1.6% for consultants vs. 2.3% for trainees; *p* = 0.349) and recurrent reflux (1.4% for consultants vs. 1.0% for trainees; *p* = 0.508)	–

A comparative study of TAPP did not find any significantly higher postoperative complication rates or recurrence rates for patients operated on by supervised trainees compared with those operated on by experienced surgeons (62).

Likewise, a comparable recurrence rate was identified for open and laparoscopic inguinal hernia surgery performed by trainees under supervision of consultants (53).

Only in the operative time was a difference found in a further study of the Lichtenstein operation to the disadvantage of trainees (63). But not even a longer operative time was found for trainees compared with consultants in all studies (64, 65).

For more complex procedures, such as hiatoplasty and fundoplication for reflux disease, high demands are made on the trainees in preparation for such operations as well as on the supervisor in order to assure comparable outcomes (66).

The available studies clearly demonstrate that consistent supervision by experienced consultants of trainees play a pivotal role in mastering the learning curve. This virtually eliminates the negative implications of the trainee learning curve for the patient.

Somewhat longer trainee operative times are not a problem for the patient but rather are just a matter of higher costs (67). But what is essentially more important is the aspect of patient safety. Each trainee should be supervised by an experienced consultant until they have mastered the learning curve for the respective procedure. The fact that, as stated above, the learning curve in hernia surgery procedures can be very long (TEP) means a considerable investment in training young surgeons. Furthermore, a sufficiently large caseload must be available for training. This also calls for the formation of a certain number of centers in hernia surgery with specified caseloads (68).

Annual caseload specifications for individual hospitals and each surgeon have important implications for the patient outcome. This is now discussed below on the basis of the available literature.

## Surgeon volume

An overview of systematic reviews has shown strong evidence of an association between higher volumes and better outcomes in surgery (69).

An analysis of inpatient Hospital Episode Statistics in 125,342 patients with inguinal hernia repair showed for surgeons with a low laparoscopic hernia repair caseload an increased reoperation rate (70).

In a registry-based analysis of 16,240 laparo-endoscopic (TEP, TAPP) primary inguinal hernia repairs low-volume surgeons (< 25 procedures per year) have significantly higher recurrence and pain on exertion rates than high-volume surgeons (≥25 procedures per year) (71).

Another study also confirmed the link between a high surgical volume and improved outcome for TEP repair of inguinal hernia (72).

In a study comparing the results of surgeons with an annual volume of > 30 vs. 15–30 vs. < 15 TEP repairs the perioperative complication and recurrence rates were lowest in the high volume group (73).

An analysis from the New York Statewide Planning and Research Cooperative System with 18,047 patients found a strong association between individual surgeon incisional hernia repair volume (< 36 vs. ≥36 repairs/year) and reoperation rates, operative efficiency, and charges (74). The authors concluded that preferential referral to high-volume surgeons may lead to improved outcomes and lower costs (74).

In a study of the National Impatient Sample patients treated at high—volume hospitals with >60 ventral hernia repairs per year were less likely to experience a major complication (OR 0.88; 95% CI 0.82–0.96; *p* = 0.002) or wound-based complication (OR 0.84; 95% CI 0.76–0.92; *p* < 0.001) (75). The authors concluded, that hospitals performing larger numbers of ventral hernia repairs, despite caring for a more complex patient population, may be associated with better patient outcomes than lower volume hospitals (75).

## Discussion

In addition to the well-known influence factors that impact the outcome in hernia surgery, the influence of the surgeon under different aspects can be demonstrated. That gives rise to a debate about appropriate training in hernia surgery (7). Studies have indicated that surgical trainees are not receiving sufficient experience (8). Traditionally, surgery has been taught and learned through a structured training program and proctorship (76). The orthodox apprenticeship approach of surgical training where trainees learn from their supervisors is no longer sustainable (76). This longstanding training approach is being increasingly challenged by legal and ethical concerns for patient safety, working time regulations, the cost, and surgical complications (77). As direct consequence of these challenges the interest in simulation-based training concepts has increased dramatically (78). In systematic reviews simulation-based training courses contribute to a shortening of the learning curve and improvement of trainee's surgical skills (21, 22, 78). This reduces the perioperative complication rate during the learning curve (24). Despite the advantages simulation-based training courses have not been fully incorporated into surgical training curriculum (78). But experts expect that this will become reality over the next decade (78). The Association of Surgeons in Training recommends for training units the introduction and funding of local hospital-based skills labs with appropriate training and simulation equipment (26). In a pilot project for improving surgical training the Royal College of Surgeons of England has integrated simulation-based training courses for developing surgical skills earlier, so that time is not wasted, particularly in the early years of surgical training (79). Since laparo-endoscopic and advanced open hernia operations are complex procedures, trainees should definitely participate in well-structured, 2 days training courses with theoretical and practical training on simulations (7, 25) and anatomic specimens before they perform their first procedures on a patient.

Supervision by experienced surgeons is another important aspect for prevention of perioperative complications and avoidance of the negative impact of the learning curve of the surgeon in training when performing hernia surgery procedures. Consistent supervision of trainees in the learning curve can achieve perioperative complication rates and long-term outcomes on a par with those of an experienced consultant, thus completely eliminating the negative effects of the trainee learning curve on the patients. However, this presupposes the availability of an adequate number of experienced consultants to engage in supervision. This also demonstrates that training in surgery is associated with considerable costs but that investment is crucial to patient safety. However, each surgery-teaching/training hospital should allocate funds in its budget to cover these costs.

Qualification of the training consultants is another important aspect. The studies presented above demonstrate the clear link between the annual caseload per consultant and the outcome. Accordingly, the consultants supervising trainees when performing surgical procedures should themselves conduct a minimum number of the most important hernia surgery procedures each year. That calls for a certain degree of specialization in hernia surgery.

The aspects comprising the learning curve, supervision, and surgeon volume are relevant arguments that support specialization and formation of centers in hernia surgery for optimal implementation of the aforementioned requirements (80). This presupposes the availability of a corresponding caseload and specialization of consultants in hernia surgery. Developments to that effect should be based within departments of general surgery and cover the entire range of hernia surgery because the infrastructure of a large hospital is needed for complex hernia surgery.

## Author contributions

FK literature search, literature analyses, publication concept, publication draft.

### Conflict of interest statement

The author declares that the research was conducted in the absence of any commercial or financial relationships that could be construed as a potential conflict of interest.
